# Supplementary material for: Validation of a Prospective Urinalysis-Based Prediction Model for ICU Resources and Outcome of COVID-19 Disease: A Multicenter Cohort Study
Source: J Clin Med. 2021 Jul 9;10(14):3049. doi: 10.3390/jcm10143049 (PMC8306631; doi:10.3390/jcm10143049)
Supplement: Supplementary file 1 [file jcm-10-03049-s001.zip › S1_UMG_Covid-19_assNephritis_StudyProtocol_V1.1.pdf]

Study Title: Covid-19 associated Nephritis as early predictor for complicated course of disease

Protocol Code: UMG_Co19-Nephritis

EudraCT Number: 2020-001798-60
ClinicalTrials.gov Identifier: NCT04347824

Protocol

Version: 1.1 Date: 15.05.2020

Sponsor of the Observational Study

Universitätsmedizin Göttingen
Georg-August-Universität Göttingen Stiftung Öffentlichen Rechts

Coordinating Principal Investigator (LKP)

Prof. Dr. med. Oliver Gross

- Confidential -

The information contained in this protocol has to be kept strictly confidential. Therefore the protocol is only provided to Investigators in confidence for review, to study staff, Independent Ethics Committee/Institutional Review Board, regulatory authorities, CROs, and to obtain written informed consent from patients/parents/legal guardian.

2 Approval Signature Page

The following persons accept the content of this protocol and confirm to conduct this study in compliance with Good Clinical Practice and applicable regulatory requirements.

Approved by:

	**15. Mai 2020**
Prof. Dr. Oliver Gross<br>**Coordinating Principal Investigator**	**Date**

The present study protocol was prepared in accordance with the relevant ICH-GCP criteria.

3 Organisational Structure

Sponsor
Universitätsmedizin Göttingen
Georg-August-Universität Göttingen
Robert-Koch-Str. 40
37075 Göttingen

Coordinating Principal Investigator (LKP)
Prof. Dr. Oliver Gross
Klinik für Nephrologie und Rheumatologie
Universitätsmedizin Göttingen
Robert-Koch-Str. 40
37075 Göttingen

Leading Ethics Committee
Ethikkommission der Universitätsmedizin Göttingen
Vorsitzender: Prof. Dr. med. Jürgen Brockmöller
Von-Siebold-Str.3
37075 Göttingen

Medical Statistics
Prof. Dr. Tim Friede
Abt. Medizinische Statistik
Universitätsmedizin Göttingen
Humboldtallee 32
37073 Göttingen

Steering Committee

Göttingen:	Prof. Dr. Sabine Blaschke, Prof. Dr. S. Scheithauer, Prof. Dr. O. Moerer, Prof. Dr. O. Gross, Prof. Dr. Tim Friede
Aachen:	Prof. Dr. Jürgen Floege
Hamburg:	Prof. Dr. Tobias Huber
Köln:	Dr. Matthias Kamm

Clinical Project Management / Trial Office
Prof. Dr. Oliver Gross
Klinik für Nephrologie und Rheumatologie
Universitätsmedizin Göttingen (UMG)
Robert-Koch-Str. 40
37075 Göttingen
Tel.: +49 (0) 551 39 22925
Fax: +49 (0) 551 39 65977
gross.oliver@med.uni-goettingen.de

Trial Project Management
Universitätsmedizin Göttingen
Ressort Forschung und Lehre
Studienzentrum UMG

Von-Bar-Str. 2/4
37075 Göttingen
Tel.: +49 (0) 551 39 60812
Fax: +49 (0) 551 39 60846
E-Mail: studienzentrum-umg@med.uni-goettingen.de

Trial Sites:
A list of Trial Sites participating in this trial is on file at the Trial Office, Universitätsmedizin Göttingen (UMG)

Emergency Contact

Prof. Dr. Oliver Gross
Klinik für Nephrologie und Rheumatologie
Universitätsmedizin Göttingen
Robert-Koch-Str. 40
37075 Göttingen
Tel. (office): +49 (0) 551 39 65309
Fax (office): +49 (0) 551 39 65977
E-Mail: gross.oliver@med.uni-goettingen.de

Private address:
Prof. Dr. Oliver Gross
Schildweg 25
37085 Göttingen
Tel. (home): +49 (0) 551 7896800

4 Table of Contents

1 Study Title: Covid-19 associated Nephritis as early predictor for complicated course of disease 1
2 Approval Signature Page 2
3 Organisational Structure 3
4 Table of Contents 5
5 Synopsis 6
5 Study Synopsis in German 11

5 Synopsis

**Study Title**	**Covid-19 associated Nephritis as early predictor for complicated course of disease**
**Type of Project**	Non-interventional, observational retrospective (and in parts prospective) study in patients with Covid-19 infection to assess the impact of the associated nephritis.on systemic disease progression, complications and need for ICU.
**Sponsor**	Georg-August-Universität Göttingen<br>Stiftung Öffentlichen Rechts<br>Universitätsmedizin Göttingen<br>Vorstand<br>Robert-Koch-Str. 42<br>37075 Göttingen<br>Tel.: +49 (0) 551 39-61001<br>Fax: +49 (0) 551 39-61002
**Coordinating Principal Investigator**	Prof. Dr. Oliver Gross<br>Klinik für Nephrologie und Rheumatologie<br>Universitätsmedizin Göttingen<br>Robert-Koch-Str. 40<br>37075 Göttingen<br>Tel.: +49 (0) 551 39 65309<br>Fax: +49 (0) 551 39 65977<br>Email: gross.oliver@med.uni-goettingen.de
**Deputy for Coordinating Principal Investigator**	Dr. Jan Böckhaus<br>Klinik für Nephrologie und Rheumatologie<br>Universitätsmedizin Göttingen<br>Robert-Koch-Str. 40<br>37075 Göttingen<br>Tel.: +49 (0) 551 39 65309<br>Fax: +49 (0) 551 39 65977<br>Email: jan.boeckhaus@med.uni-goettingen.de
**(Sub-) Investigators**	A list of sub-investigators participating in this study is kept at the Trial Office (UMG, Göttingen)

**Participating Trial Sites, Data Management and Responsibilities**	A list of investigators and trial sites participating in this study are kept on file at the Trial Office (UMG, Göttingen).<br>Local Principal Investigators will be<br>1) University Hospital Aachen: Prof. Jürgen Floege<br>2) University Medical Center Hamburg-Eppendorf: Prof. Tobias Huber<br>3) Cologne General Hospital and University Witten-Herdecke: Prof. Achim Jörres<br>4) University Medical Center Göttingen: Prof. Oliver Gross<br>Principal Investigators will have the responsibility that no personal data will be reported to the Trial Office of the Coordinating Principal Investigator.<br>Pseudonymised Data using standardized questionnaires will be sent to the Trial Office by fax (or pdf-scans).<br>ICH-GCP conform data management will be provided by the local team of the Coordinating Principal Investigator, Tanja Albrecht-Nock and Jan Böckhaus, using the four-eyes principle.
**Hypothesis**	Covid-19 associated nephritis is a - possibly overlooked - predictor for adverse outcome, which can be easily screened for by a simple and inexpensive urine-analysis. If confirmed, the findings would allow early prediction of later need for ICU-capacity, better allocation of patients for clinical trials, and preventive strategies focused on the capillary leak (nephrotic like) syndrome including treatment, which can save lives. Same might apply for risk-evaluation of outpatients.
**Rationale**	Parameters predicting risks for Covid-19 patients are urgently sought. The urine-status might be an easy way to detect systemic capillary leak and to early predict fluid overload, respiratory failure, later need for ICU, and death.<br>Observational data of the first Covid-19 patients at the UMG helped to generate an algorithm, which allows early detection of Covid-associated nephritis and to quantify the risk for respiratory decompensation by capillary leak syndrome (Figure).<br>Why does Covid-19 cause nephritis and how does this predict complications? Podocytes express ACE2; Covid-19 has recently been located to podocytes and tubular cells with a nephritis-like histology. Other zoonoses, such as Hanta-virus, cause nephrotic syndrome inducing cardiopulmonary syndrome. Life-threatening complications of nephrotic syndrome similar to capillary leak syndrome are well known as are preventive therapies.
**Study Medication**	Non-interventional observational trial
**Placebo**	Non-interventional observational trial

**Study Design**	Strictly observational, non-interventional data acquisition with retrospective data analysis.

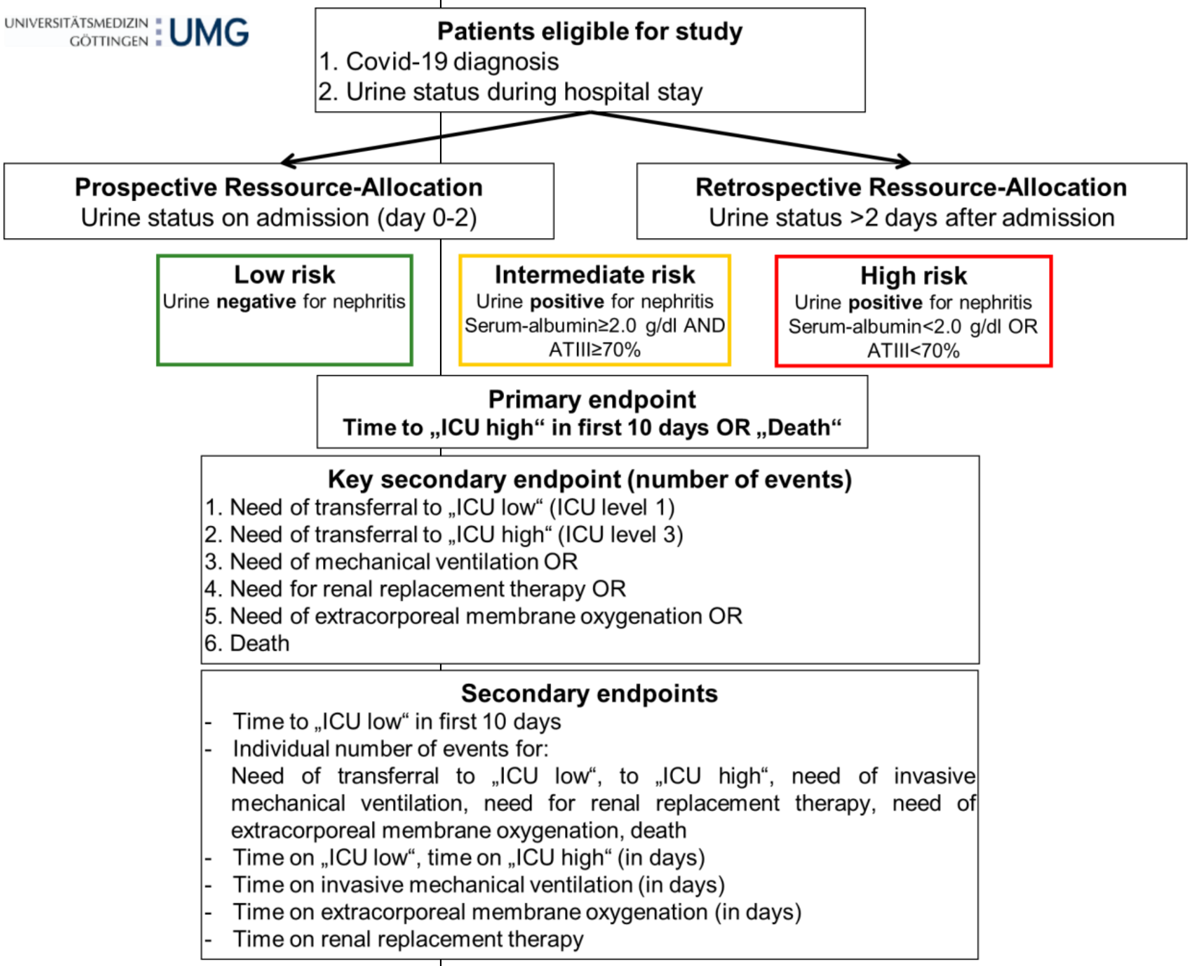

Patients will be devided into three groups:
I. low risk

This group has a normal urine status.

Abnormal urine status is defined anuric OR as 2* or more of the following findings:
1. urine osmolarity below normal values
2. leukozyturia
3. hematuria
4. albuminuria/ proteinuria

* if urine is positive for nitrite or bacteria, abnormal urine status is defined as 3 or more of the findings.

II. intermediate risk
This group has an abnormal urine status on admission to hospital
WITH serum-albumin >= 2.0 g/dl AND
WITH antithrombin III level >= 70%.

	III. high risk<br>This group has an abnormal urine status on admission to hospital PLUS<br>serum-albumin < 2.0 g/dl OR antithrombin III level < 70%.
**Timetable**	Date of study / recruitment Start April2020<br>Date of termination of recruitment July 2020<br>Date of study termination December 2020
**Total Number of Patients**	Total: minimum 100, up to 250
**Study population**	Patients with Covid-19 infection
**Inclusion Criteria**	1. approved Covid-19 diagnosis (by PCR, CT-scan).<br>2. urine status during hospital stay.<br>3. Patient expressed willingness to participate in observational studies during hospital stay.
**Exclusion Criteria**	1. uncertain Covid-19 diagnosis;<br>2. lack of urine status during hospital stay<br>3. Patient expressed unwillingness to participate in observational studies during hospital stay.
**Documentation Schedule**	One documentation needed at end of hospital stay.
**Study Objectives**	**Study Objectives:**<br>This study aims to determine, if the urine-status on admission can predict disease-aggravation based on the hypothesis that an abnormal urine status at admission is an early sign of systemic capillary leak in Covid-19 infection. This study also aims to determine, if the urine-status on admission can predict fluid overload, respiratory failure, later need for ICU, and death.<br><br>**Study Endpoints:**<br>**Primary Endpoint:** Time to event defined as<br>Time to transferral to ICU (ICU level high) OR Death*<br><br>* in the first 10 days after admission to hospital<br><br>**Key Secondary Endpoint:** number of events<br>1. Need of transferral to „ICU low“ (ICU level 1)*<br>2. Need of transferral to „ICU high“ (ICU level 3)*<br>3. Need of mechanical ventilation* OR<br>4. Need for renal replacement therapy* OR<br>5. Need of extracorporeal membrane oxygenation* OR<br>6. Death

	* in the first 10 days after admission to hospital<br><br>**Secondary endpoints**<br><br>- Time to „ICU low“ in first 10 days<br>- Time on „ICU low“ (in days),<br>- Time on „ICU high“ (in days),<br>- Time on invasive mechanical ventilation (in days)<br>- Time on extracorporeal membrane oxygenation (in days)<br>- Time on renal replacement therapy (in days)<br>- lowest serum-albumin<br>- lowest antithrombin III
**Safety**	Safety is assessed by the recording of adverse events and laboratory parameters.
**Statistical Analysis**	Please see Statistical Analysis Plan for details.<br><br>In brief, statistical analysis of this study will be the responsibility of the Dept. Medical Statistics, University Medical Center Göttingen, Georg-August-Universität.<br><br>The primary endpoint ‘Time to first event will be assessed. If indicated, additional potentially confounding variables such as age, sex and kidney function will be added to the model.
**Pharmacological-toxicological Evaluation**	Not applicable
**Risks, Adverse Drug Reactions, Drug Interactions, Restrictions, Contraindications, Procedures in Case of Emergency**	Not applicable
**Risk-benefit Analysis**	Not applicable

6 Study Synopsis in German

**Studien-Titel**	**Covid-19 assoziierte Nephritis als Frühmarker für einen komplizierten Krankheitsverlauf**
**Projekttyp**	Eine nicht-interventionelle retrospektive (teils prospektive) Beobachtungsstudie bei Patienten mit Covid-19 Infektion zur Evaluation der Bedeutung der assoziierten Nephritis für das Fortschreiten der Systemerkrankung, für Komplikationen und für die Notwendigkeit einer künftigen Intensivversorgung.
**Sponsor**	Georg-August-Universität Göttingen<br>Stiftung Öffentlichen Rechts<br>Universitätsmedizin Göttingen<br>Vorstand<br>Robert-Koch-Str. 42<br>37075 Göttingen<br>Telefon: +49 (0) 551 39 61001<br>Telefax: +49 (0) 551 39 61002
**Leiter der Beobachtungsstudie**	Prof. Dr. Oliver Gross<br>Klinik für Nephrologie und Rheumatologie<br>Universitätsmedizin Göttingen<br>Robert-Koch-Str. 40<br>37075 Göttingen<br>Tel.: +49 (0) 551 39 65309<br>Fax: +49 (0) 551 39 65977<br>E-Mail: gross.oliver@med.uni-goettingen.de
**Vertreter des Leiters der Beobachtungsstudie**	Dr. Jan Böckhaus<br>Klinik für Nephrologie und Rheumatologie<br>Universitätsmedizin Göttingen<br>Robert-Koch-Str. 40<br>37075 Göttingen<br>Tel.: +49 (0) 551 39 65309<br>Fax: +49 (0) 551 39 65977<br>Email: jan.boeckhaus@med.uni-goettingen.de
**(Sub-) Prüfer**	Eine Liste beteiligter Sub-Prüfer dieser Studie liegt dem Studienzentrum (UMG) Göttingen vor.
**Beteiligte Studienzentren, Daten Management und Verantwortlichkeiten**	Eine Liste beteiligter Studienzentren liegt dem Studienzentrum (UMG) Göttingen vor.<br>Als Hauptprüfer/Studienleiter (Local Principal Investigators) sind vorgesehen:<br>1) Universitätsklinik Aachen: Prof. Jürgen Floege<br>2) UKE Hamburg: Prof. Tobias Huber<br>3) Kliniken der Stadt Köln, Krankenhaus Köln-

	Merheim, Universität Witten-Herdecke: Prof. Achim Jörres<br>4) Universitätsmedizin Göttingen: Prof. Oliver Gross<br><br>Die jeweiligen Hauptprüfer tragen vor Ort die Verantwortung, dass dem Studienzentrum Göttingen keine personenbezogenen Daten übermittelt werden. Die pseudonymisierten Daten werden mittels standardisiertem Fragebogen an das Studienzentrum per Fax (oder pdf-Scan) übermittelt.<br>Ein ICH-GCP konformes Datenmanagement wird durch das lokale Studienzentrum in Göttingen (unter Aufsicht des LKPs) durch Frau Tanja Albrecht-Nock und Herrn Jan Böckhaus mittels Vier-Augen-Prinzip gewährleistet.
**Hypothese**	Wir wollen herausfinden, ob die Diagnose einer Covid-19 assoziierten Nephritis helfen kann, den Gesamtverlauf der Erkrankung, Komplikationen und Intensivaufenthalte vorherzusagen. Der Urinstatus bei Aufnahme/ bei Covid-19 Diagnose könnte so als einfach zu erhebendes Frühwarnzeichen dazu dienen, Risiko-Patienten früher zu erkennen und zu behandeln.
**Begründung**	Viele Covid-19 Patienten scheinen einen auffälligen Urinstatus haben, der an andere Zoonosen wie Hanta-Viren erinnert. Zudem zeigen besonders die schwer erkrankten Patienten Zeichen eines capillary leak Syndroms (ähnlich wie nephrotisches Syndrom) mit Hypalbuminämie, Antithrombin III Mangel und Proteinurie. Da Podozyten ACE2 exprimieren, erscheint es wahrscheinlich, dass die Niere bei Covid-19 auch miterkranken kann.
**Studienmedikation**	Entfällt.
**Placebo**	Entfällt.
**Studiendesign**	Streng rein observatorische, nicht-interventionelle Beobachtungstudie mit einer retrospektiven Daten-sammlung und -auswertung.<br><br>Die Patienten werden in drei Risikogruppen unterteilt:<br><br>I. Niedriges Risiko:<br>Diese Gruppe hat bei Aufnahme ins Krankenhaus einen normalen Urinstatus.<br><br>Ein nicht-normaler (auffälliger) Urinstatus ist definiert als anurisch ODER 2* oder mehr der folgenden Befunde:

	1. erniedrigte Urin-Osmolarität<br>2. Leukozyturie<br>3. Hämaturie<br>4. Albuminurie/ Proteinurie<br><br>*Wenn der Urinstatus Bakterien oder Nitrit-positiv ist, gilt der Urinstatus erst bei 3 der folgenden Befunde als auffällig.<br><br>II. Intermediäres Risiko:<br>Diese Gruppe hat bei Krankenhausaufnahme einen auffälligen Urinstatus<br>UND Serum-Albumin ≥ 2,0 g/dl UND<br>Antithrombin III Spiegel ≥ 70%.<br><br>III. Hohes Risiko:<br>Diese Gruppe hat bei Krankenhausaufnahme einen auffälligen Urinstatus UND<br>Serum-Albumin < 2,0 g/dl ODER<br>Antithrombin III Spiegel < 70%.

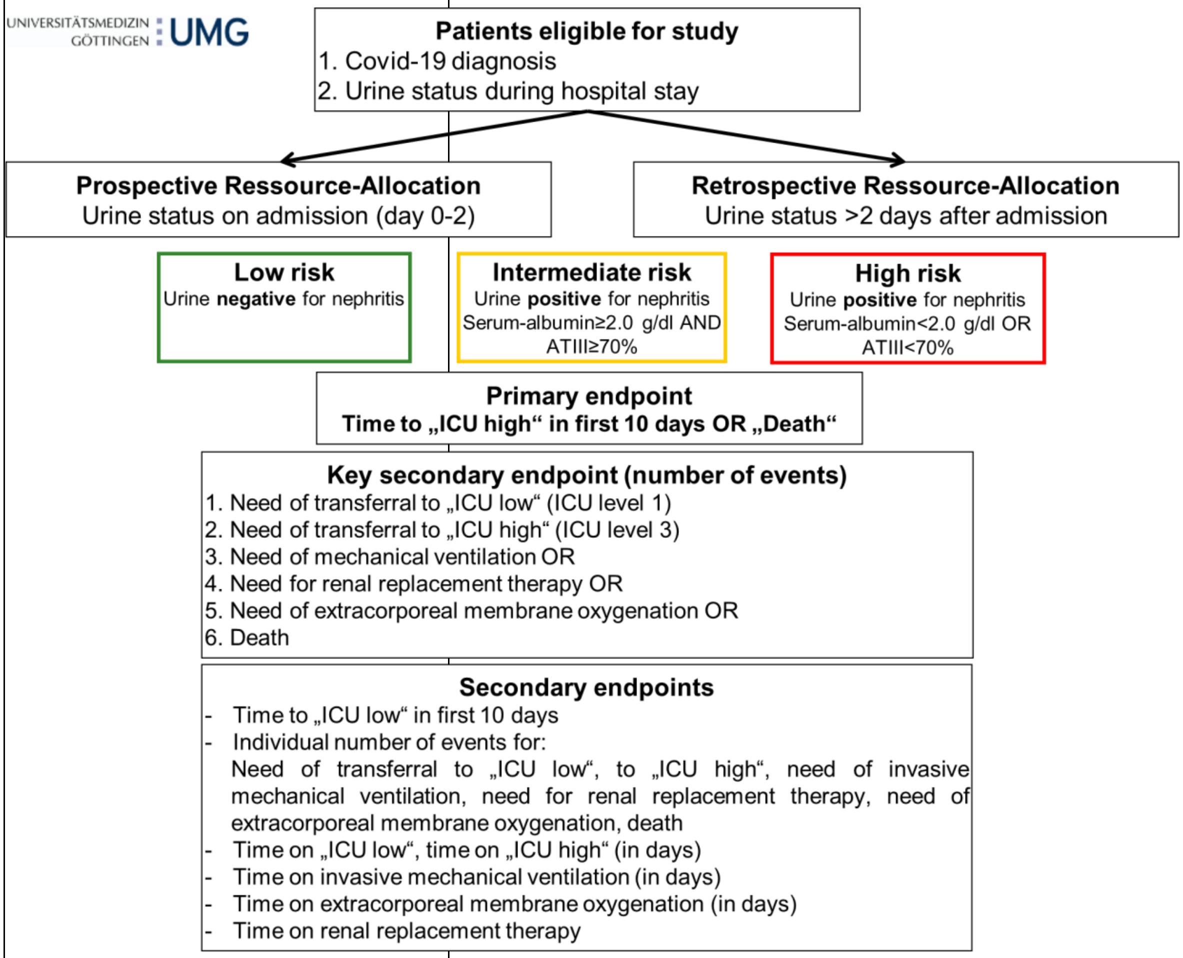

**Zeitplan**	Beginn der Studie April 2020<br>Ende der Rekrutierung Juli 2020<br>Ende der Studie Dezember 2020
**Anzahl der Patienten, gesamt**	Total: Minimum 100, bis zu 250
**Studienpopulation**	Patienten mit Covid-19 Erkrankung
**Einschlusskriterien**	1. Diagnose von Covid-19 (mittels PCR, CT-Thorax) oder dringender klinischer Verdacht;<br>2. Urinstatus während des Krankenhausaufenthaltes.<br>3. Einwilligung des Patienten zur Teilnahme an der Beobachtungsstudie
**Ausschlusskriterien**	1. unsichere Covid-19 Diagnose;<br>2. fehlender Urinbefund während des Krankenhausaufenthaltes<br>3. Fehlende Bereitschaft des Patienten zur Teilnahme an der Beobachtungsstudie.
**Visen- und Dokumentations-Plan**	Einmalige Dokumentation während des Krankenhausaufenthaltes.
**Studienziele**	**Studienziel:**<br>Die Studie soll herausfinden, ob ein auffälliger Urinbefund bei Aufnahme helfen kann, den Gesamtverlauf der Covid-19 Erkrankung, Komplikationen und Intensivaufenthalte vorherzusagen.<br><br>**Studien-Endpunkte:**<br>**Primärer Endpunkt:** Zeit bis Verschlechterung definiert als:<br><br>Notwendigkeit der Verlegung auf Intensivstation ODER Tod<br><br>**Haupt Sekundärer Endpunkt:**<br>Anzahl an Ereignissen:<br>1. Notwendigkeit der Verlegung auf IMC,*<br>2. Notwendigkeit der Verlegung auf ICU,*<br>3. Erfordernis der mechanischen Beatmung,* ODER<br>4. Erfordernis der Nierenersatztherapie* ODER<br>5. Erfordernis der extrakorporalen Membran-oxygenierung (ECMO)*<br>6. Tod<br><br>*in den ersten 10 Tagen<br><br>Sekundäre Endpunkte:<br><br>- Zeit bis Verlegung auf IMC in den ersten 10 Tagen

	Individuelle Anzahl von Ereignissen in den ersten 10 Tagen:<br>1. Notwendigkeit der Verlegung auf IMC,<br>2. Notwendigkeit der Verlegung auf ICU<br>3. Erfordernis der mechanischen Beatmung, ODER<br>4. Erfordernis der Nierenersatztherapie<br>5. Erfordernis der extrakorporalen Membran-oxygenierung (ECMO)<br>6. Tod<br><br>- Zeit auf IMC (in Tagen)<br>- Zeit auf ICU (in Tagen)<br>- Beatmungszeit (in Tagen)<br>- Zeit an ECMO-Therapie (in Tagen)<br>- Zeit an Dialysetherapie (in Tagen)<br>- niedrigstes Serum-Albumin<br>- niedrigstes Antithrombin III.
**Sicherheit**	Die Sicherheit wird anhand der dokumentierten Ereignisse und Laborparameter evaluiert.
**Kriterien für Therapieabbruch**	Entfällt.
**Statistische Analyse**	Die statistische Auswertung erfolgt unter der Verantwortung der Abteilung Medizinische Statistik, Universitätsmedizin Göttingen, Georg-August-Universität. Details zur statistischen Analyse sind im statistischen Analyseplan festgelegt.<br>Der primäre Endpunkt „Zeit bis Event" wird ausgewertet werden. Wenn angezeigt, werden bei der Auswertung zusätzliche Variablen wie Alter, Geschlecht und Nierenfunktion berücksichtigt.
**Pharmakologische und toxikologische Analyse**	Nicht Teil der Studie
**Risiken, Unerwünschte Medikamenten-Reaktionen, Vorgehen bei unerwünschten Ereignissen**	Nicht Teil der Studie
**Nutzen-Risiken Analyse**	Nicht Teil der Studie
