# Supplementary material for: Validation of a Prospective Urinalysis-Based Prediction Model for ICU Resources and Outcome of COVID-19 Disease: A Multicenter Cohort Study
Source: J Clin Med. 2021 Jul 9;10(14):3049. doi: 10.3390/jcm10143049 (PMC8306631; doi:10.3390/jcm10143049)
Supplement: Supplementary file 1 [file jcm-10-03049-s001.zip › S2_UMG_Covid-19_assNephritis_StatisticalAnalysisProtocol_V1.1.pdf]

UNIVERSITÄTSMEDIZIN GÖTTINGEN UMG

Statistical Analysis Plan

Version: 1.1 Date: 15 JUN 2020

Covid-19 associated nephritis as early predictor for complicated course of disease

UMG_Co19-Nephritis

**Study Protocol**	Version 1.1 dated 15 MAY 2020
**EudraCT No.**	2020-001798-60
**Clinicaltrials.gov-No.**	NCT04347824
**Internal Protocol ID No.**	UMG_Co19-Nephritis
**SAP Version**	1.1/ 15 JUN 2020
**SAP Revisions**	*In comparison to Version 1.0 the following was added: The association of COVID-19 associated nephritis and disease severity will be assessed in cross-sectional analyses.*
**Sponsor**	University Medical Center Göttingen<br>represented by the Head of Clinical trials unit UMG<br>Robert-Koch-Str. 40<br>37075 Göttingen, Germany
**Coordinating Investigator**	Prof. Dr. med. Oliver Gross<br>Department of Nephrology and Rheumatology, UMG<br>Robert-Koch-Str. 40<br>37075 Göttingen

Approval of the Statistical Analysis Plan

Covid-19 associated nephritis as early predictor for complicated course of disease

EudraCT No.: 2020-001798-60

Protocol Version No: 1.1 dated 15 MAY 2020

Coordinating Investigator
Prof. Dr. med. Oliver Gross

15 June 2020

Date Signature

Biostatistician
Prof. Dr. Tim Friede

15 JUN 2020

Date Signature

Table of Contents

Statistical Analysis Plan 1
Approval of the Statistical Analysis Plan 2
Table of Contents 3
List of Abbreviations 4
1 Introduction 5
2 Study methods 7
3 Statistical Principles 7
4 Trial population 8
5 Analysis 9
6 References 10

List of Abbreviations

AE	Adverse Event
AMG	Medicinal Products Act / German Drug Law (*Arzneimittelgesetz*)
BID	twice a day
BMI	Body Mass Index
BP	Blood Pressure
CI	Confidence Interval
Covid-19	coronavirus disease 2019
HR	Hazard Ratio
ICU	Intensive Care Unit
WHO	World Health Organization

1 Introduction

Very recently the new pathogen severe acute respiratory syndrome coronavirus 2 (SARS-CoV-2) was identified and the coronavirus disease 2019 (Covid-19) declared a pandemic by the World Health Organization (WHO). Parameters predicting risks for Covid-19 patients are urgently sought.

1.1 Background and Rationale

The urine-status might be an easy way to detect systemic capillary leak and to early predict fluid overload, respiratory failure, later need for ICU, and death.

Observational data of the first Covid-19 patients at the UMG helped to generate an algorithm, which allows early detection of Covid-associated nephritis and to quantify the risk for respiratory decompensation by capillary leak syndrome (Gross et al, 2020).

Why does Covid-19 cause nephritis and how does this predict complications? Podocytes express ACE2; Covid-19 has recently been located to podocytes and tubular cells with a nephritis-like histology. Other zoonoses, such as Hanta-virus, cause nephrotic syndrome inducing cardiopulmonary syndrome. Life-threatening complications of nephrotic syndrome similar to capillary leak syndrome are well known as are preventive therapies.

1.2 Objectives and Endpoints

This study aims to determine, if the urine-status on admission is associated with disease severity and can predict disease-aggravation based on the hypothesis that an abnormal urine status at admission is an early sign of systemic capillary leak in Covid-19 infection. This study also aims to determine, if the urine-status on admission can predict fluid overload, respiratory failure, later need for ICU, and death.

1.3 Primary objective and endpoint

Abnormal urine status is defined anuric OR as 2 or more (if urine is positive for nitrite or bacteria, abnormal urine status is defined as 3 or more of the findings) of the following findings:

1. urine osmolarity below normal values
2. leukozyturia
3. hematuria
4. albuminuria/ proteinuria

If one normal and one abnormal urine analyses are available on two seperate days at admission to hospital, the urine status will be defined as abnormal.

Following Gross et al (2020) patients will be devided into three risk groups:

I. low risk: This group has a normal urine status.

II. intermediate risk: This group has an abnormal urine status on admission to hospital WITH serum-albumin >= 2.0 g/dl AND WITH antithrombin III level >= 70%.

III. high risk: This group has an abnormal urine status on admission to hospital PLUS serum-albumin < 2.0 g/dl OR antithrombin III level < 70%.

The primary objective is to evaluate this algorithm for risk stratification, which is prespecified and published by Gross et al (2020), in particular low risk vs. intermediate / high risk. Primary endpoint for prediction is the time from hospital admission to transferral to ICU (ICU level high) or death, whatever occurs first. For the association of the risk group obtained by the algorithm above with disease severity, the primary endpoint is the type of ward (normal, intermediate care / ICU level 1, ICU level 3) the patient is cared for at the time of urine sampling.

1.4 Secondary objectives and endpoints

The secondary objectives include the evaluation of the algorithm for risk stratification by Gross et al (2020) considering intermediate vs. high risk in terms of the primary endpoint. Furthermore, low risk vs. intermediate / high risk and intermediate vs high risk will be evaluated with regard to the following key secondary endpoint: Time to first event (whatever occurs first) out of the following list:

1. Need of transferral to „ICU low“ (ICU level 1)*
2. Need of transferral to „ICU high“ (ICU level 3)*
3. Need of mechanical ventilation* OR
4. Need for renal replacement therapy* OR
5. Need of extracorporeal membrane oxygenation* OR
6. Death

* in the first 10 days after admission to hospital

Considering low risk vs. intermediate / high risk and intermediate vs. high risk, the following secondary endpoints will be analysed:

- Components of the key secondary endpoint
- Individual number of events during the first 10 days for:
    - Need of transferral to „ICU low“, to „ICU high“, need of invasive mechanical ventilation, need for renal replacement therapy, need of extracorporeal membrane oxygenation, death
- Time on invasive mechanical ventilation (in days)
- Time on extracorporeal membrane oxygenation (in days)
- Time on renal replacement therapy

1.5 Exploratory objectives and endpoints

Among the exploratory objectives of this study is an assessment of the prognostic value of the proposed risk algorithm in addition to established prognostic factors such as age, sex and comorbidities including kidney function.

2 Study methods

2.1 Trial design

This is a prospective cohort study.

2.2 Sample Size

As per study protocol, the aim is to recruit at least 100 patients and at most 250 patients into the study. This is justified by the following considerations.

The primary comparison will be between low risk and intermediate / high risk patients with regard to the primary endpoint, i.e. time to ICU transferral or death. The expectation is that only 20% of patients will be in the low risk group whereas 80% will be in the intermediate or high risk groups. The risk for a primary outcome event is considered to be around 25% (at Day 10) in the low risk group and 50% in the combined intermediate / high risk group (relative risk (RR) of 2). Under these assumptions a total sample size of 183 (240) patients yields a power of 80% (90%) at the usual two-sided significance level of 5%. With a risk of 30% in the low risk group and otherwise same assumptions (in particular RR=2), a total sample size of 130 (172) patients yields a power of 80% (90%).

For the consideration of intermediate risk vs high risk, only 80% of the sample size can be used. The ratio of intermediate risk patients to high risk patients is considered to be 3. With risks of 40% in the intermediate risk group and 70% in the high risk group (at Day 10), a total sample size of 112 (148) patients yields a power of 80% (90%) at a two-sided significance level of 5%.

A larger sample will be available to study the association of the risk groups as determined by the algorithm proposed by Gross et al (2020) with disease severity in a cross-sectional analysis, which will provide sufficient power.

The sample size calculations were carried out using nQuery Version 8.

2.3 Statistical Interim Analyses

No interim analyses are planned.

3 Statistical Principles

3.1 Confidence intervals and p-values

Hypotheses will be tested at the usual two-sided significance level of 5%. 95% confidence intervals will be provided. No adjustments for multiple testing will be carried out.

3.2 Analysis populations

For the purpose of studying the association of the risk groups as determined by the algorithm proposed by Gross et al (2020) with disease severity all patients with a valid urine sample and the necessary demographic and clinical data will be included. For the purpose of prediction of disease

aggravation, the primary analysis population includes all patients with urine status with 2 days from hospital admission who are not directly admitted from ICU to ICU (for example by transferral from the ICU of another hospital). Patients with urine status later than 2 days from hospital admission will be included in supporting analyses only.

4 Trial population

4.1 Screening data

Screening data were not collected.

4.2 Eligibility

Inclusion criteria:

- approved Covid-19 diagnosis (by PCR, CT-scan) or suspected clinical diagnosis
- urine status during hospital stay
- Patient expressed willingness to participate in observational studies during hospital stay

Exclusion criteria:

- uncertain Covid-19 diagnosis;
- lack of urine status during hospital stay
- Patient expressed unwillingness to participate in observational studies during hospital stay
- Patient who served for the generation of the algorithm

4.3 Recruitment

Recruitment and patient flow will be depicted in a CONSORT flow diagram.

4.4 Withdrawal/follow-up

Premature study discontinuations (also known as study withdrawal or dropout) is likely to be very rare in this hospitalized population, in particular given the short follow-up time. Nevertheless, the final statistical study report will include information on timing of and reasons for any premature study discontinuations.

4.5 Baseline patient characteristics

Baseline demographics and clinical characteristics will be summarized using appropriate statistics such as frequencies and percentages for categorical data, and means and standard deviations for continuous data. The distributions of continuous variables will also be displayed as box plots. The demographic and clinical baseline characteristics include age, sex, body mass index (BMI), comorbidities such as kidney function, and albumin as well as antithrombin measurements. The baseline characteristics will be presented for the whole study population as well as stratified by

the three risk groups. Here both analysis sets will be considered, for the association with disease severity (cross-sectional study) and for the prediction of disease aggravation.

5 Analysis

5.1 Analysis methods

To assess the association of the risk groups as determined by the algorithm proposed by Gross et al (2020) with disease severity, a frequency table of the risk groups and the type of ward (normal, intermediate care / ICU level 1, ICU level 3) will be provided. The association will be formally tested by a chi-square test. A two-sided p-value will be reported.

For prediction of disease aggravation, the primary endpoint time from hospital admission to transferral to ICU or death (whatever comes first) will be analysed using a Cox proportional hazards model with the risk groups as independent variable. The primary null hypothesis to be tested is that hazard ratio (HR) for low risk vs. intermediate / high risk is larger or equal to one, i.e. H_0: HR ≥1. The hazard ratio will be reported with 95% confidence interval and p-value (Wald-type test). The performance of the prognostic model will be evaluated using the C-index and calibration slope. Both will be reported with 95% confidence intervals. These analyses will be repeated with all three risk categories (low, intermediate and high) in all patients as well as with intermediate risk vs. high risk in this subgroup of patients (excluding low risk patients). The data will be visualized by Kaplan-Meier curves stratified by risk group.

As the prognostic model is prespecified (Gross et al, 2020), this study is an external validation and all patients will be included in the validation. Supporting analyses will explore whether the algorithm could be improved, e.g. by changing thresholds for the variables used or by including additional variables. For these analyses 10-fold cross-validation will be used to adjust the results.

In supporting analyses, additional prognostic variables will be added to the models. These will include age, sex, presence of any comorbidity (e.g. kidney function, diabetes, cardiovascular disease, chronic respiratory disease), and BMI (<20, 20-30, >30 (and potentially additionally >35, if a sufficient number of patients would be in this category)).

The analyses of the key secondary endpoint will follow the same lines as the analysis of the primary outcome. The same is true for secondary endpoints including death as an outcome event. The analyses of the other secondary outcomes will need to account for death as competing event. These will be visualized by cumulative incidence functions (Aalen-Johansen estimator).

5.2 Missing data

The extent of missing data in baseline and follow-up data (outcomes) will be summarized by providing the frequencies and percentages of missing data points. Missingness due to competing events will be dealt with by using appropriate multi-state models (competing risks methodology). Missing data in baseline variables might be dealt with by multiple imputation if justified.

5.3 Harms

The primary and secondary endpoints listed include a number of clinical events related to patient safety.

5.4 Statistical software

The analyses will be carried out using SAS statistical software Version 9.4 or higher, or the R package Version 3.6.3 or higher.

6 References

Gross O, Moerer O, Weber M, Huber TB, Scheithauer S (2020) COVID-19-associated nephritis: early warning for disease severity and complications? Lancet 395: e87-e88.
